# A method for extracting small water bodies based on DEM and remote sensing images

**DOI:** 10.1038/s41598-024-51346-7

**Published:** 2024-01-08

**Authors:** Qingzhen Sun, Jinguang Li

**Affiliations:** 1 School of Civil Engineering and Architecture, Zhengzhou University of Aeronautics, Zhengzhou, 450046 China; 2 School of Water Conservancy and Transportation, Zhengzhou University, Zhengzhou, 450001 China

**Keywords:** Ecology, Environmental sciences

## Abstract

Accurate and comprehensive extraction of water bodies facilitates the study of watershed changes and water quality monitoring and also provides a reference for ecological evolution. When extracting tributaries and small water bodies based on remote sensing images, there are problems such as uncertain threshold selection, more edge voids, and extraction difficulties. The improved water body extraction method (Combination Normalized Difference Water Index), fuses Digital Elevation Model and remote sensing images. The river network is extracted from the Digital Elevation Model and the remote sensing image is normalized. After fusion, threshold comparison is performed, and the threshold of the river network location is used as the basis for connecting the broken water bodies, it is compared and verified with the normalized thresholds to achieve accurate identification and complete extraction of water bodies. Taking the water bodies in Xinyang City as an example, the overall accuracy of the confusion matrix was the highest, with an overall accuracy of 93.0% compared with the traditional index method. Combined with water bodies in Zhengzhou City for model validation, it has high applicability. Whether the water body is large or small, the extraction results are complete, with no voids or broken, and the accuracy is high.

## Introduction

Remote sensing imagery, with its advantages of large coverage, rich spectral information, high spatial and temporal resolution, abundant data sources, and low cost, plays an important role in the rapid extraction of water bodies and water quality monitoring. Rapid extraction of basic information on large water bodies using remote sensing satellite imagery has been an efficient and reliable application technique. After decades of development, the current water body extraction methods can be divided into three categories according to the principle: single-band method^1^, multi-band inter-spectral analysis method^2^, and water body index method. With the application of machine learning and neural networks, the water body extraction field has also proposed remote sensing water body extraction methods based on machine learning methods^3^ and neural networks^4,5^ in recent years^6^.

The index water body extraction methods in practical production applications are mainly based on the spectral characteristics and spectral differences of different features in each band. It uses the spectral difference transform to enhance the differences between the water body and other background features, to maximize the distinction between the water body and the background features from the remote sensing image. The single-band threshold method selects a specific band in the remote sensing image where the difference between the water body and other background features is the largest, then extracts the water body information by setting the appropriate threshold. This method is simple, but only applicable to flat areas, and has been replaced by the multi-band inter-spectral analysis method. The classical multiband methods include Normalized Difference Water Index (NDWI)^7–9^, Modified Normalized Difference Water Index (MNDWI)^10–12^, and Revised Normalized Difference Water Index (RNDWI)^13–15^.

NDWI uses near-infrared and visible bands to construct the index formula, which better suppresses the vegetation information, but buildings, shadows, mountains, and clouds all have a large impact on the results, so NDWI is only suitable for flat areas^16,17^. Based on NDWI, infrared bands are added to obtain MNDWI enhances the feature differences between water bodies and buildings, but it is still disturbed by mountains and shadows^18^. RNDWI can reduce the interference of mountain shadows and weaken the influence of mixed pixels on the accuracy of the extraction results^19^. Both NDWI and MNDWI are unable to differentiate between fine water bodies such as urban rivers, and both of them are affected by the insufficient information of mixed pixels, and they can't separate the boundaries of these kinds of water bodies; RNDWI is easy to confuse the water body with the river bank, and it performs unstable for the extraction of the water body edges. The interpolation threshold method can better reduce the effect of mountain shadows on water body extraction in mountainous areas^20^. The exponential model performs poorly in mountainous areas and cannot extract details of narrow and long linear rivers in small watersheds^21^. The water body index method based on band operation easily mis-extracts water body areas due to the interference of actual topography, shadows, and other features, and cannot solve the difficulties of small watersheds with fine water bodies and poor extraction results of water body edges.

Water body extraction methods based on machine learning and neural networks need to accumulate a large amount of sample data for repeated model training. And they have higher requirements for parameter setting and adjustment. Although it can improve the comprehensiveness and accuracy of water body information extraction, the overall process is complex, low real-time, and cannot fully meet the real-time and efficient application requirements in water body monitoring practice.

At present, most of the water body extraction work based on remote sensing images focuses on large rivers, main streams, and other large water bodies; while the extraction process of small tributaries and narrow water bodies is relatively complicated, and the extraction difficulty is even greater than that of large open water bodies, the integrity and accuracy of water body extraction are difficult to ensure, and the boundary of the water body is fragmented and the problem of voids is serious.

Based on the problems of inland tributaries, narrow river water bodies with smaller areas, shallow water depth, and difficulty in extracting edges, resulting in easy misclassification of water body features and serious breakage of extraction results, this paper proposes an improved method: the water body fusion extraction method Combination Normalized Difference Water Index (CNDWI), fuses Digital Elevation Model (DEM) and remote sensing images. Through the spectral characterization of Landsat-8 OLI correlation bands of water bodies and background features, the appropriate combination of bands is selected to effectively distinguish water bodies and background features. Meanwhile, the regional features of water bodies were accurately identified in the DEM, and detailed information on the river network in the region was extracted, which served as the basis for identifying fine water bodies and connecting broken water bodies. Combining the river network data with the water body information in the remote sensing images achieves the purpose of improving the accuracy and complete extraction of fine water bodies and boundaries.

The high-precision extraction of minor water bodies expands the monitoring range of water quality remote sensing inversion, guarantees the extraction of large area water bodies while maximizing the complete extraction of fine water bodies, expands more fine water body information for remote sensing water quality monitoring, and improves the accuracy and completeness of remote sensing water quality inversion. In this study, we are committed to improving the water body extraction method to enhance the accuracy and completeness of water body extraction in remote sensing images, especially in the extraction of narrow and fine water bodies showing higher accuracy. At the same time, the extraction results in large water bodies are comprehensive and accurate, and the extraction results perform as well as the traditional water body index methods. This improvement provides richer water body information for water body extraction and remote sensing water quality inversion, which is conducive to finer water quality monitoring work. In addition, in urban environments, the shadows of buildings and vegetation have a greater impact on water body extraction, which increases the difficulty of the extraction work. In this paper, we propose a new water body extraction method: Combination Normalized Difference Water Index (CNDWI), which utilizes the fusion of remote sensing imagery and DEM for water body extraction to minimize the problem of crippled and fragmented extraction results, and to further reduce the difficulty of analyzing the urban fine water body system comprehensively. The proposed method focuses on the study of small elongated water bodies, such as tributaries, and at the same time performs well in the parallel monitoring of large and small water bodies, which provides a solid foundation for the comprehensive study of watersheds.

## Results

The improved remote sensing image-based extraction method for small water body extraction, CNDWI, selects Landsat-8 OLI blue, green, and two shortwave infrared bands by band analysis. Characteristic differences between water bodies and other features are enhanced by band operations, and the edges of fine water bodies as well as background-rich water bodies are extracted quickly and accurately, with good resistance to the effects of mountainous areas and vegetation shadows. The fusion of DEM to extract river network information is used to further integrate the results from partially fragmented water bodies, enabling CNDWI to extract complete water bodies in complex water bodies. The total accuracy of water body classification at CNDWI sampling sites is up to 93.0%. The advantages of the CNDWI method compared with the traditional water body index method are (1) Good extraction effect. It effectively suppresses the background information, distinguishes the water body from the shadow of buildings and mountain vegetation, and extracts the edges of the water body completely without voids. (2) Complete edge extraction. It is sensitive to the information of fine water bodies, accurately recognizes water bodies, and reduces the phenomenon of misjudgment and wrong extraction. The integration of the river network information obtained from the digital elevation model can ensure that the water body information is fully recognized, and the integrity of the water body extracted from the remote sensing image is better and more accurate. (3) Focus on small water bodies. Whether it is a tributary outside the city or a narrow river inside the city, the extraction results of traditional methods are poor, but CNDWI can supplement the detailed information extracted from large water bodies and provide better data support for the comprehensive study of water. (4) Stronger applicability. For regional differences, remote sensing image differences, and water environment differences, CNDWI extraction results are accurate and have good applicability.

## Discussion

### Water extraction results

The experiment is based on the extraction of the central water bodies in the administrative area of Xinyang City, and the background features of the experimental area are mainly vegetation, buildings, and settlements, ENVI and ArcGIS are used for data processing and model validation. In addition, a comparison experiment with the traditional index methods NDWI, MNDWI, and RNDWI is conducted to test the accuracy and applicability of the extraction method of water bodies in this paper.

**Figure 1:**
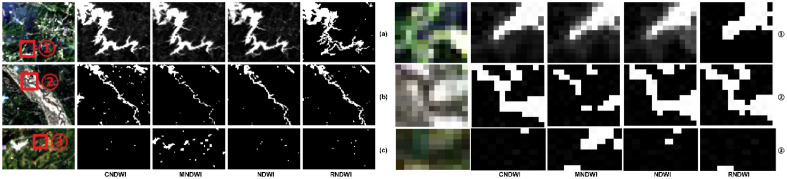
Extraction results and details of reservoir edges, small rivers, and mountain shadows.

The water bodies in the experimental area are mainly reservoirs and small rivers. The fine parts of water bodies with typical characteristics are selected for display. Reservoirs in the basin (a) have a large water body area, and many tributaries contain fine water bodies and larger tributaries, the edge of the water body is complex, showing a serious irregular distribution, and the edge of the extracted water body is representative. The fine water body (b) belongs to the narrow tributary, the water body has a long fine section, and there are diversion and merging phenomena, the distribution of the water body is more complicated, and it has the characteristics of the narrow water body. The shadow of the mountain is easy to be misjudged as a water body, and the selection of the mountain shadow (c) without a water body area can accurately reflect the extraction results of the shadow, and verify whether there is a possibility of misjudging the shadow as a water body. The binarization results of different methods to extract the edge of reservoirs and small rivers are shown in Fig. 1.

It shows the water extraction results of the reservoir in (a), and the red box ① shows the details of the reservoir edge. The topography at the edge of the reservoir is mostly mountainous and forested. The details of box ① show that CNDWI water body extraction edges are continuous without holes, similar to the edges of satellite images; water body index methods NDWI, MNDWI and RNDWI water body extraction of the reservoir edge have different degrees of voids, and breaks, especially the MNDWI and RNDWI have serious voids, and the NDWI extraction results are incomplete and much smaller than the actual water body area. It shows the extraction results of fine tributary water bodies in (b), and the red box ② shows the tributary details. Box ② Detail shows that CNDWI, NDWI, and RNDWI water bodies were extracted continuously, and MNDWI water bodies were extracted with voids and breaks. CNDWI combined with the information supplement of the river centerline, the water body extraction results are more complete than the traditional index method, and there is no false river direction caused by mixed image pixels. It shows the water body extraction results in the shadowed area of the mountain in (c), and the red box ③ shows the details of the shadowed part. The details of box ③ show that the traditional water body index MNDWI, NDWI, and RNDWI misjudged the shadow of the mountain as a broken water body, so the shadows will be output as a water body, especially MNDWI's misjudgment results are serious, and a large number of shadows will be extracted as a water body; NDWI and RNDWI misjudgment is less, and a minority of erroneous extraction results; CNDWI does not have the phenomenon of misjudgment, and the extraction results are the most accurate.

The above experimental results show that CNDWI has complete extraction results for open water bodies (reservoirs) with large areas. It can accurately distinguish the boundary between the water body and the background features, and precisely extract large water bodies. When extracting water bodies in narrow tributaries, the water bodies have good continuity without broken and fractured phenomena, and it is capable of extracting tiny tributary water bodies completely. It can effectively identify mountain shadows and water bodies, as much as possible minimize misjudgment resulting in wrong output results, suppress the influence of background features on water body extraction, and provide more complete data support for remote sensing water quality research. The river network extracted by DEM further integrates some of the broken water body extraction results to ensure that the results are not seriously affected when the threshold value fluctuates within a certain range, which reduces the difficulty of threshold selection. Overall, compared with the traditional water body index extraction results, CNDWI not only performs well in large water areas, but also in tiny tributaries, and the integrity and accuracy of the extracted water bodies are higher.

### Accuracy evaluation

**Figure 2:**
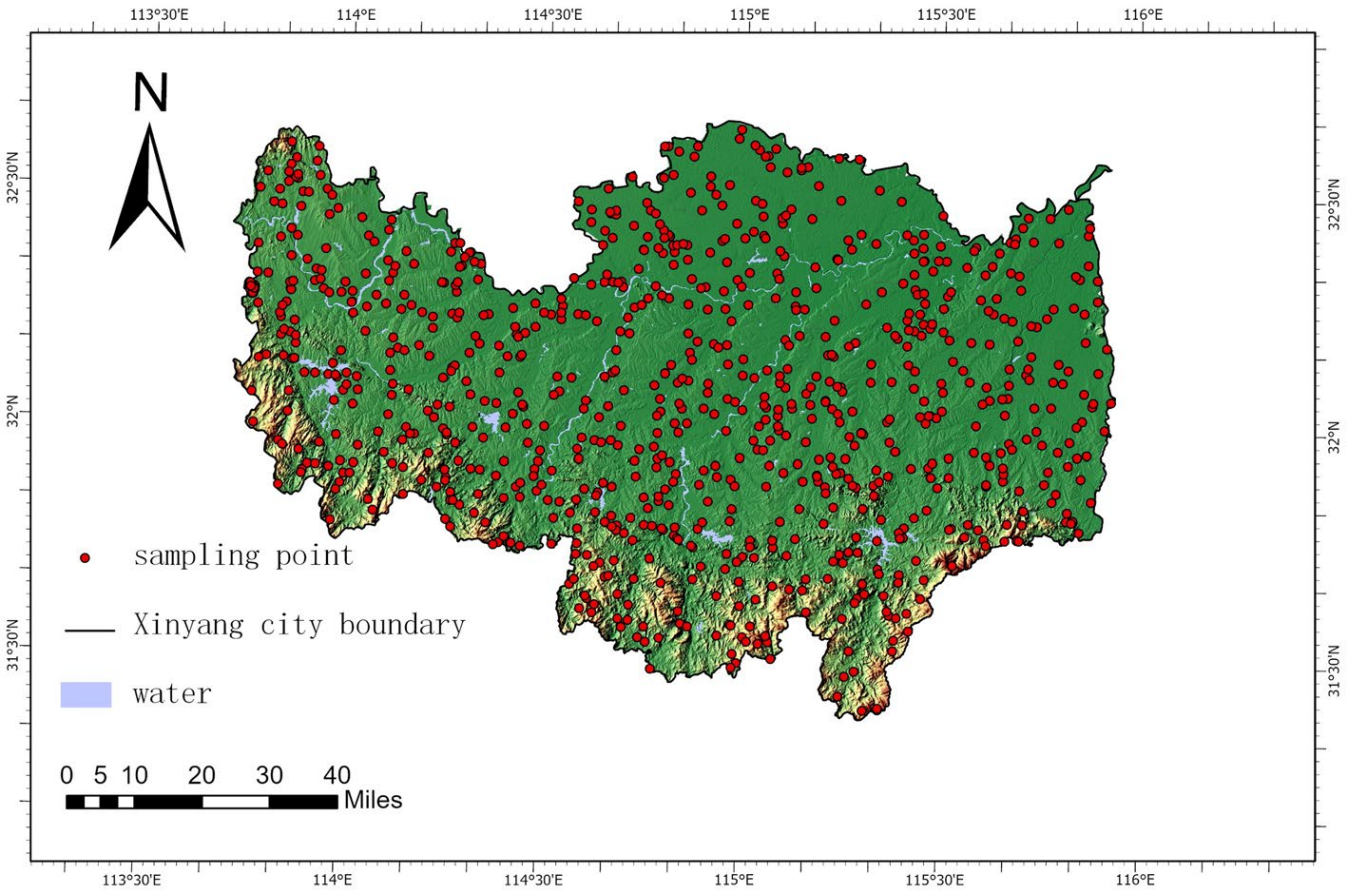
Distribution of sampling points. (The data source for Fig. 2 is public image: https://www.91weitu.com/. The processing software is: v.19.3.4 Build By 91 weitu, and ArcGIS).

To objectively verify the accuracy of the CNDWI water body index, 867 ground points were randomly selected in Xinyang administrative area as shown in Fig. 2. The random arrangement of sampling points to verify the accuracy, intercepting remote sensing images according to the administrative boundaries of Xinyang City, using ArcGIS to generate random sampling points, and superimposing the location information of the sampling points onto the remote sensing images of Landsat 8, to realize the random setup of the sampling points, and to avoid the influence of human factors on the verification of the accuracy of the sampling points. According to the original remote sensing images and field conditions, the real data of all sampling points are categorized into water-body sampling points and non-water-body sampling points. The real geomorphology is marked as 1 if it is a water body and 0 if it is not a water body. It is determined whether the location of the sampling points is a water body or not in the results of the water body index calculation, and then it is interactively verified with the real geomorphology of the sampling points. According to the extraction results of different water body indices, ArcGis10.5 was used to determine whether the sampling point was discriminated as a water body calibrated attribute value by the index method. Classification as a water body was labeled as 1 and non-water body was labeled as 0. The overall accuracy of different water body extraction methods was compared by calculating the confusion matrix of the classification results.

Confusion matrix is a commonly used accuracy evaluation method in statistics. Four basic indicators were obtained by calculating the model data in the samples, namely TP correctly predicts positive: the extraction result is a water body and the real landform is a water body; FN incorrectly predicts negative: the extraction result is a water body and the real landform is a non-water body; FP incorrectly predicts positive: the extraction result is a non-water body and the real landform is a water body; and TN correctly predicts negative: the extraction result is a non-water body and the real landform is a non-water body.

The kappa coefficient k of the confusion matrix is an integral representation of the accuracy of the classification result as shown in the equation (1),

1 $$ k = \frac{{\mathop P\nolimits_{o} - \mathop P\nolimits_{e} }}{{\mathop {1}\nolimits_{{}} - \mathop P\nolimits_{e} }} $$

In the formula, $\mathop P\nolimits_{o}$ is the overall classification accuracy, $\mathop P\nolimits_{e}$ is [Positive (TP) + Positive (FP)] ∗ [Positive (TP) + Negative (TP)] divided by the "square of the number of samples".

The results of the NDWI, MNDWI, RNDWI, and CNDWI confusion matrices are shown in Table 1. The CNDWI has the best performance in terms of overall accuracy and confusion matrix—the kappa coefficient of k is 0.802, and the total accuracy of sampling point classification is 93.0%. The improved CNDWI method effectively solves the problem of difficult extraction of water body edges and small water bodies, and the classification accuracy of sampling points is higher.

**Table 1:** Accuracy of different water body index methods.

Water extraction methods	NDWI	MNDWI	RNDWI	CNDWI
Waterbody production accuracy	0.841	0.973	0.904	0.953
Non-water body production accuracy	0.901	0.736	0.854	0.803
Water user accuracy	0.963	0.919	0.950	0.937
Non-water user accuracy	0.647	0.902	0.742	0.890
Total accuracy	0.856	0.915	0.892	**0.930**
Kappa coefficient	0.655	0.754	0.721	**0.802**

Significant values are in bold.

### Validate model

There are differences in water bodies and remote sensing images in different regions, and there are many narrow and small water bodies in the city with complex distribution, the water body extraction results are seriously affected by buildings and shadows, so the urban water bodies are selected off-site for method validation, to check the applicability and accuracy of this method.

#### Urban water extraction

**Figure 3:**
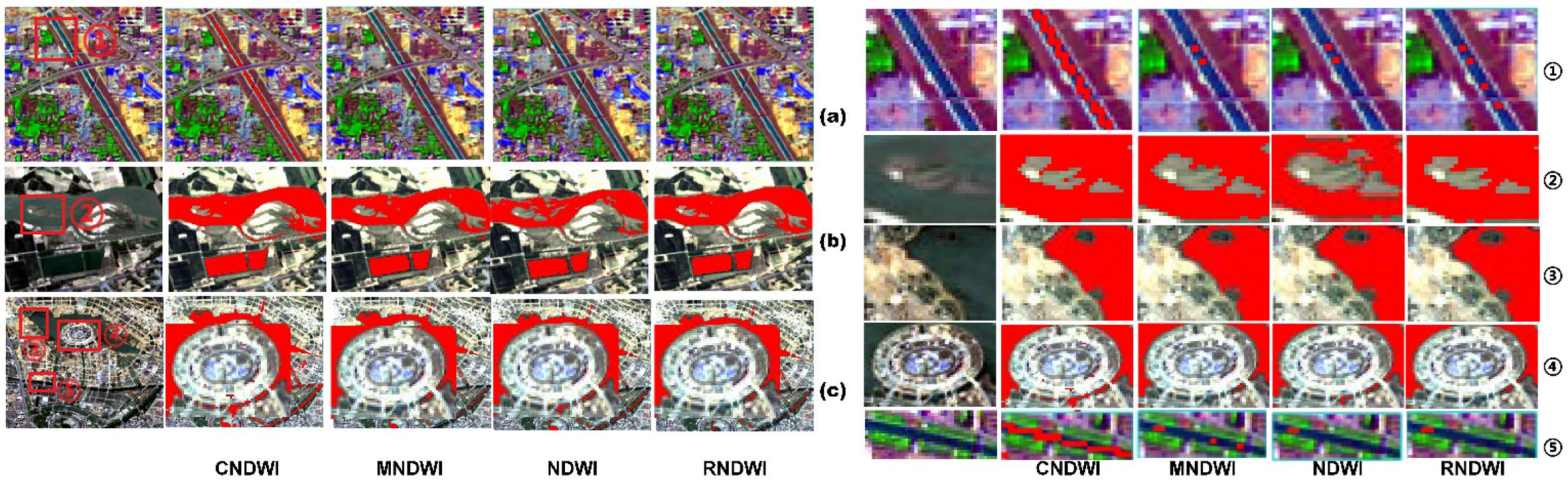
Results of water extraction and the details of partial water.

Landsat-8 OIL imagery of Zhengzhou City on April 28, 2017, was selected for off-site water body validation, and the water body extraction results of the four methods are shown in Fig. 3. Each row shows the original remote sensing imagery, CNDWI, MNDWI, RNDWI, and NDWI water body extraction results in order from left to right. According to the characteristic differences of various water bodies, boxed ① to ⑤ detail areas are urban small and medium-sized rivers, part of the Yellow River water bodies, Longhu Lake near the wetland park area water bodies, Longhu Lake surrounded by the construction area, and Jinshui River and Hongqi Canal confluence water bodies, respectively.

The Yellow River ② and the Long Lake ③ have similar results. The performance of the four water body index methods is the same, for the larger open water, all the water body indexes are stable, and the extraction results are comprehensive. CNDWI and the traditional water body index methods have the same results, which apply to the extraction of water bodies in different regions and can be applied to meet the requirements of the extraction of a wide range of water bodies. As shown in boxed ③, some small-area water bodies in the yellow circle are located in the interior of the wetland park, with more vegetation and shadows, MNDWI is unable to extract the information of water bodies in this location, while CNDWI, RNDWI, and NDWI can distinguish between vegetation shadows and water bodies, and accurately extract the information of water bodies in this location. All the methods in boxed ④ have similar results in extracting the edges of large water bodies in Longhu Lake, but RNDWI misjudges some water bodies affected by buildings and incorrectly extracts the results of water bodies. MNDWI, NDWI, and CNDWI are better at distinguishing the differences between water bodies and buildings, accurately identifying the area of water bodies affected by buildings, and avoiding the misjudgment of water bodies and landforms. Boxed ① and ⑤ are narrow river channels in different areas of the city, and the pixels occupied by this channel are mostly mixed pixels in the remote sensing images. The CNDWI extraction results of the river in boxed ① are better, with the least missing parts and the richest water body information, which provides a good basis for remote sensing water quality research. However, RNDWI and NDWI perform similarly, with some small water bodies unrecognizable and a certain degree of water body fragmentation. MNDWI performs the worst, with broken and fractured water bodies that cannot support water quality monitoring and remote sensing water quality inversion research. In boxed ⑤, CNDWI performs better than RNDWI, with no fracture phenomenon in the yellow circled area, MNDWI performs relatively poorly, with serious water body fracture, but NDWI performs the worst, which is almost unable to effectively extract the information of the river water body. In general, the CNDWI method has stable performance and the best results for the extraction of small water bodies and has similar stability and completeness for the extraction of water bodies in large areas as the traditional water body index extraction method. Based on ensuring that fine water bodies can be completely extracted by the CNDWI method, fusing the river network information obtained from the digital elevation model, and connecting the few broken water bodies that are still discontinuous, more complete and continuous water body extraction results can be obtained.

#### Validation analysis

Compared with traditional methods of water body extraction from remote sensing images, CNDWI has similar operational steps in urban complex water body extraction, but the extraction results are more comprehensive and accurate compared with traditional index methods. Unlike the distribution of water bodies outside the city in the watershed, the distribution of water bodies in the city is complex, with more narrow and small water bodies, and most of them are shallow, and the shadows of the surrounding buildings and vegetation have a greater impact on the extraction of water bodies. CNDWI can accurately distinguish the water bodies from the background features, recognize the difference between the shaded part and the water bodies, and accurately extract the water bodies in the long, narrow, and shallow river channels. In addition, the river network data can connect the broken water bodies, which makes the water body extraction results more complete.

Combined with the water body extraction results in Xinyang City and Zhengzhou City, it is obvious that CNDWI has the best performance. The CNDWI method is not only capable of extracting large open water bodies, but also small water bodies in tributaries and urban distribution, and it is adaptable to extracting water bodies from remote sensing images. It performs well in water environments with different geographical areas, geomorphology, and water quality, especially the accuracy and completeness that meet the needs of remote sensing water quality monitoring, which is conducive to doing water research work with better accuracy.

## Methods

### Normalized difference water index method

The classical water body extraction index methods NDWI, MNDWI, and RNDWI corresponding bands are calculated in equations (2)–(4). The NDWI can better suppress the vegetation information and emphasize the water features, but the differentiation of buildings and soils is poor, and it is affected by clouds and mountain shadows. Features are considered to be waterbodies when the NDWI calculation produces positive values. The MNDWI improves the discrimination between waterbodies and urban residential areas, reduces the influence of background noise, and is more conducive to the accurate extraction of waterbodies^22^. The RNDWI uses only the shortwave infrared and red bands, which reduces the influence of the mixed image elements on the extraction results to some extent, but the stability of the extraction of the edges of water bodies is poor. The determination of the thresholds of the three methods must be different according to the characteristics of the study area and the changes in spectral reflectance, which is adjusted after manual analysis, and the water body extraction is performed after setting the appropriate thresholds.

2 $$ NDWI = {{(G - NIR)} \mathord{\left/ {\vphantom {{(G - NIR)} {(G + NIR)}}} \right. \kern-0pt} {(G + NIR)}} $$

3 $$ MNDWI = {{(G - MIR)} \mathord{\left/ {\vphantom {{(G - MIR)} {(G + MIR)}}} \right. \kern-0pt} {(G + MIR)}} $$

4 $$ RNDWI = {{(SWIR - R)} \mathord{\left/ {\vphantom {{(SWIR - R)} {(SWIR + R)}}} \right. \kern-0pt} {(SWIR + R)}} $$

In the formula, NDWI means normalized differential water body index; G, R, NIR, MIR, and SWIR respectively donate the green, red, near-infrared, mid-infrared, and short-wave infrared bands of remote sensing satellite imagery.

### River network identification

**Figure 4:**
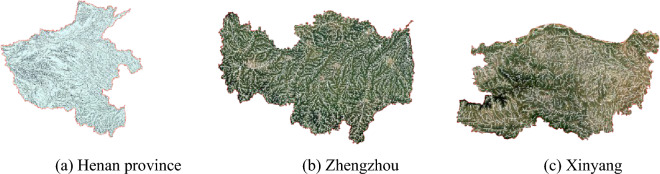
The river network in Henan province and Xinyang and Zhengzhou cities. (Original DEM data for Fig. 4 were obtained from ASF Data Search (alaska.edu). The river network extraction was performed using Arc GIS.)

The DEM (ASF Data Search (alaska.edu)) expresses the surface morphology, effectively identifies the water body information, and forms the river network data, which facilitates the water body identification in the remote sensing image. The density of the river network is affected by the size of the screening flow, the smaller the screening flow, the denser the river network, and the fine water bodies are shown in the river network^23^. Narrow water bodies and fine tributaries are shown in the river network, which provides data support for the identification of fine water bodies and the process of connecting broken water bodies in remote sensing images. The river network in Henan province and Xinyang and Zhengzhou cities is shown in Fig. 4. The river network shown in the figure reflects the tributaries and narrow water bodies information, which provides basic data for the CNDWI model. During the river network extraction process, a buffer of 15 m was set. Considering that Landsat8 OLI contains multispectral 8 bands with 30 m spatial resolution, one panchromatic band with 15 m spatial resolution, and thermal infrared data. Fusing the 8-band 30m multispectral data and the 15m panchromatic data can get a better fusion effect with 15m resolution image data. Considering that the extracted river network data are fused with remote sensing data, a 15m buffer is set.

### Water extraction from remote sensing images

**Figure 5:**
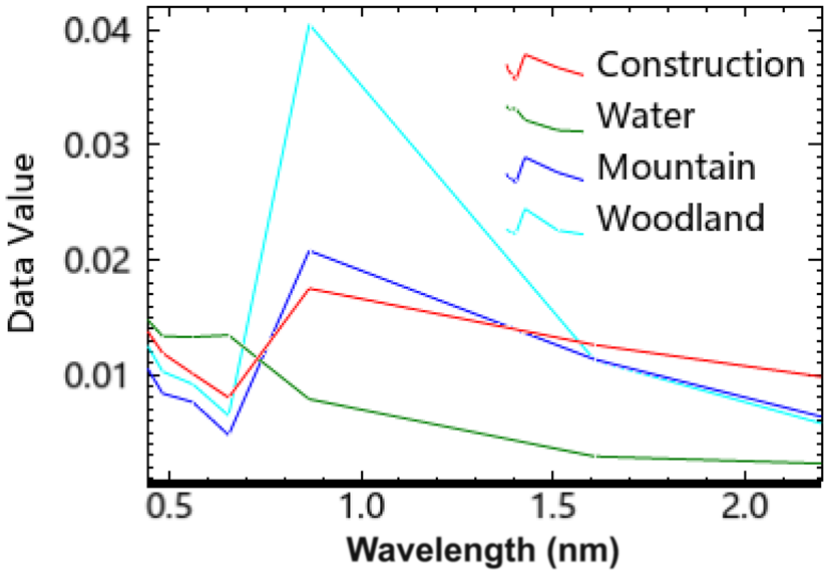
Spectral reflectance of ground objects.

Based on the band information of Landsat-8 OLI images (ceode.ac.cn), the reflectance of different features is analyzed. The band reflectance distributions of the four main types of features, namely water bodies, buildings, mountains, and forests, are shown in Fig. 5, and their horizontal coordinates are blue (0.45–0.51), green (0.53–0.59), red (0.64–0.67), near-infrared (NIR) (0.85–0.88), short-wave infrared 1 (SWIR1) (1.57–1.65), and short-wave infrared 2 (SWIR2) (2.11–2.29) bands in descending order from left to right.

Satellite remote sensing images are based on the different responses of different features to electromagnetic waves and the thermal radiation information generated by the features themselves, and the water in rivers and lakes is generally not pure, mainly as a result of the interaction between the light transmitted into the water and the chlorophyll in the water, the sediment, the depth of the water and its thermal characteristics, so that the water body is generally presented as green on the remote sensing image. Water bodies in the spectral response in general in the blue-green light band absorption rate is low, in other bands, especially the infrared band absorption rate is very high, and vegetation, mountains, and buildings in these two bands of absorption of energy are small, high reflectance, which makes the water body in these two bands and other features have a clear difference. Therefore, in remote sensing, the near-infrared band is often used to construct a model to extract information about water bodies, and the characteristics of water bodies reflected in the electromagnetic wave spectral bands are the basis for the extraction of water bodies using remote sensing technology.

As can be seen from the spectral characteristics curve in Fig. 5, the reflectivity of the water body is stronger in the green and red bands, and changes gently in the short-wave infrared band, but the reflectivity is the weakest, and the overall reflectivity of the water body is lower than that of the building. From the near-infrared band to the short-wave infrared band, the overall features of buildings, forests, and mountains have similar trends. The maximum reflectance of water bodies in Landsat 8 satellite images is concentrated in the blue and green bands, while the two short-wave infrared bands have the lowest reflectance. More importantly, the reflectance differences of other features in the blue, green, and two short-wave infrared bands are much smaller than that of water bodies^24^. Therefore, we can use band manipulation to increase the feature differences between water bodies and other background features, effectively differentiate water bodies in remote sensing images, and make water body extraction results more accurate.

Compared with other features in the blue-green band and two short-wave infrared bands with smaller differences, the difference in reflectivity of the water body is stronger, according to the principle of the water body index model, through the wave operation of the remote sensing image, the interference of the buildings, mountain shadows, and vegetation is suppressed to enhance the difference between the characteristics of the water body and the features, and then the $\mathop {NDWI}\nolimits_{F}$ is obtained by normalization through the waveform operation, as in Eq. (5). In this paper, the blue-green band and two short-wave infrared bands are utilized to perform sum and difference operations, respectively, followed by ratio processing. The calculation results of normalization show that the size and positive–negative relationship of the ratio can be used to distinguish the water body from other features, and the band sum, difference, and ratio operation can effectively suppress the information of other features, so that the normalization result of the water body is a large positive value, and the other features are negative or smaller positive values, which distinguishes the water body from the non-water body part and improves the accuracy of water body extraction. In addition, an area threshold can be set to filter out smaller water body areas. This helps to remove inconspicuous water body areas due to noise or improper threshold setting.

5 $$ \mathop {NDWI}\nolimits_{F} = \frac{G + B - SWIR1 - SWIR2}{{G + B + SWIR1 + SWIR2}} $$

In the formula, G and B are green and blue bands, respectively, and SWIR1 and SWIR2 bands are two infrared bands. Through the transformation of the four bands, increase the band difference characteristics, and finally make the band operation results normalized, control the results between 0 and 1, which is conducive to the selection of the threshold value, and more accurate extraction of small water bodies.

### CNDWI modeling

CNDWI fusion of remote sensing images and DEM, community extraction process, the DEM extraction of the river network as the overall framework for the extraction of water bodies, the remote sensing image to identify the results of the water body, and the fusion of the river network.

**Figure 6:**
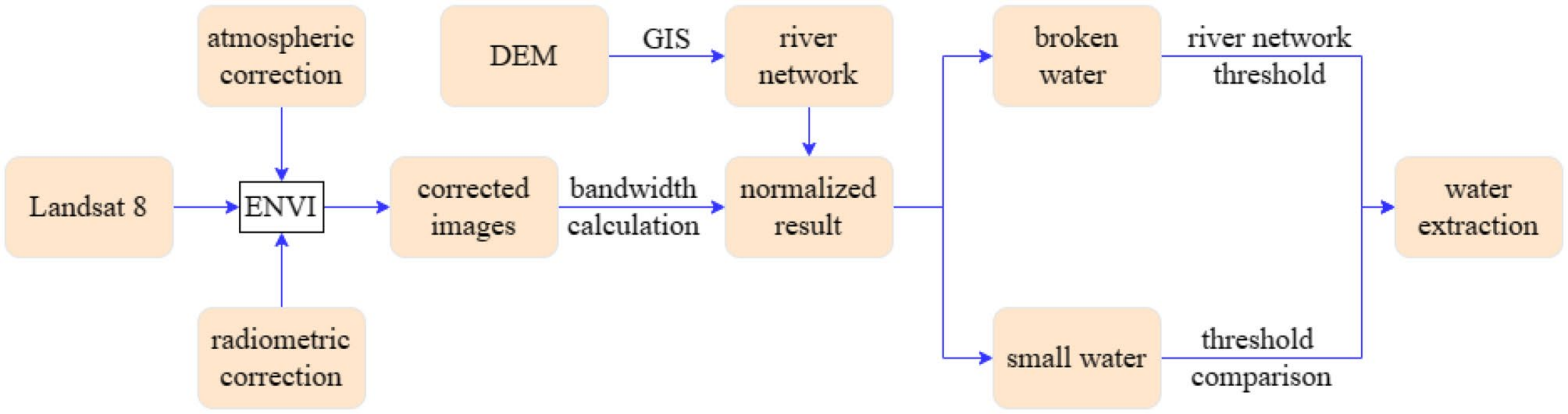
The process of water extraction.

After the superposition of the river network and the water body detected by remote sensing images, the threshold of the position occupied by the river network should be the larger positive value of the $\mathop {NDWI}\nolimits_{F}$, and the threshold of the position bodies are connected based on the river network information to reduce the lack of information caused by water body voids, thus realizing the connection of broken water bodies. Broken water bodies may be affected by buildings and shadows, and the surrounding thresholds have smaller positive or negative values. The connection of broken water bodies is realized by determining the range of larger positive values through the location of the river network. For narrow and thin river water bodies, the threshold range is compared, and the river and remote sensing extraction results are verified with each other to ensure the integrity of the water body extraction results. Overall, the river network as a prerequisite for water body extraction increases the integrity of the water body and verifies the accuracy of the extraction results. The specific steps are illustrated in Fig. 6.

Water body extraction was based on Landsat 8 remote sensing images, and the extracted water body information was imported into ArcGIS, while overlaying with the DEM extracted river network data. For larger water bodies (reservoirs, lakes, etc.), the accuracy and completeness of water body extraction can be cross-validated based on the location of the river network and the distribution of the extracted water bodies. For small water bodies (long and narrow rivers, etc.), the water bodies extracted according to Landsat 8 are mostly broken and fractured. Therefore, based on the river network location, the normalization results of the water bodies at the fracture are compared and analyzed. The $\mathop {NDWI}\nolimits_{F}$ of normalization result corresponding to the river network location is set to be greater than the threshold for distinguishing between water bodies and non-water bodies, to realize the connection between the extraction results of fractured water bodies and intact water bodies. The thresholds are adjusted according to the distribution of different types of water bodies, so that it can be more flexible to adapt to changes in the terrain and reduce the problem of disconnected water bodies.

### Supplementary Information

Supplementary Figures.

## Data Availability

All data generated or analysed during this study are included in this article and its supplementary information files.
